# Treatment of intrathoracic anastomotic leak after esophagectomy with the sump drainage tube

**DOI:** 10.1186/s13019-021-01429-7

**Published:** 2021-03-23

**Authors:** Qifan Yin, Shaohui Zhou, Yongbin Song, Xuejiao Xun, Nana Liu, Lijun Liu

**Affiliations:** Department of Thoracic Surgery, Hebei General Hospital, 348, West He-Ping Road, Shijiazhuang, 050051 Hebei Province People’s Republic of China

**Keywords:** Intrathoracic anastomotic leak, Esophagectomy, Sump drainage tube

## Abstract

**Background:**

Intrathoracic esophagogastric anastomotic leak is one of the deadliest complications after esophagectomy. In recent years, we have implemented new method for the treatment of intrathoracic esophagogastric anastomotic leak with the nasogastric placement of sump drainage tube through fistula into abscess cavity. The aim of this study is to compare the efficacy of the new method and conventional therapies for intrathoracic anastomotic leak after esophagectomy.

**Method:**

Esophagectomy and esophagogastric anastomotic procedures were performed in 875 patients at our institution from January 2008 to December 2019. Of these patients, 43(4.9%) patients developed intrathoracic anastomotic leaks postoperatively were enrolled into our study and their clinical data were retrospectively assessed. 20 (47%) patients from January 2008 to December 2012 received conventional treatments (group 1) known as the traditional “three-tube method”, and 23 (53%) patients from January 2013 to December 2019 received new treatments (group 2), consisted of conventional therapies and the nasogastric placement of sump drainage tube through fistula into abscess cavity.

**Results:**

The presence of intrathoracic anastomotic leak was proven by contrast esophagography in 43 patients (4.9%). Among them, The average duration of chest tube was 47 days in group 1 and 28 days in group 2. The average length of leak treatment was 52 days in group 1 and 35 days in group 2. The average length of postoperative hospital stay was 56 days in group 1 and 39 days in group 2, respectively. 7(35%) patients among 20 patients died from intrathoracic anastomotic leak in group 1; and 3(13%) patients among 23 patients died from intrathoracic anastomotic leak in group 2. Compared with the conventional treatments (group 1), The average duration of chest tube was significantly decreased in the new treatments (group 2) (*P* < 0.01), as well as the length of leak treatment (*P* < 0.05) and the length of postoperative hospital stay (*P* < 0.01). However, there was no significant difference in the mortality rate (*P* = 0.148 > 0.05).

**Conclusion:**

In conclusion, Our results showed this method of the nasogastric placement of sump drainage tube through fistula appears to be an safe, effective, technically feasible treatment option for intrathoracic esophagogastric anastomotic leak. The efficacy and feasibility could be further investigated with a well-designed and large-scale RCT research.

## Introduction

Esophageal cancer is one of the most common malignant tumors in China. **The new cases have reached 250,000 a year, accounting for more than half of the 480,000 cases worldwide** [1]. Radical surgery remains the optimal treatment option for resectable esophageal tumors [2]. Anastomotic leak is one of the deadliest complications to occur after esophagectomy [3, 4]. The incidence of anastomotic leak is 10–25% at the cervical region, but mortality rate in this region is low [5–7]. In the literature, the incidence of intrathoracic anastomotic leaks is 3–25% and the mortality rate is 30–60% [8–10]. Approximately 40% of general postoperative mortality rate is related to esophagogastric anastomotic leaks [11]. Intrathoracic esophagogastric anastomotic leak after esophagectomy is still a significant cause of postoperative morbidity and mortality [12]. The most effective treatment option for intrathoracic esophagogastric anastomotic leak is still controversial, and there is no standard treatment. Some surgeons recommend aggressive surgery, while others prefer conservative treatments, such as perianastomotic drainage, total parenteral nutrition, nasogastric decompression, and use of broad-spectrum antibiotics [13, 14]. With the development of interventional radiology and endoscopic therapy, we have implemented new method for the treatment of intrathoracic esophagogastric anastomotic leak with the nasogastric placement of sump drainage tube through fistula into abscess cavity. The aim of this study is to compare the efficacy of the new method and conventional therapies for intrathoracic esophagogastric anastomotic leak after esophagectomy for esophageal or **esophagogastric junction** carcinoma.

## Patients and methods

### Patients

Esophagectomy and esophagogastric anastomotic procedures were performed in 875 patients for esophageal or esophagogastric junction cancer at our institution of Hebei General Hospital from January 2008 to December 2019. Of these patients, 56 (6.4%) patients were diagnosed with anastomotic leak. Thirteen patients were excluded because of simplex cervical anastomotic leaks, who were cured by opening of the wound and daily irrigation and packing. Eventually, 43(4.9%) patients developed intrathoracic anastomotic leaks postoperatively were enrolled into our study and their clinical data were retrospectively evaluated. All of them **developed** mediastinitis and empyema. Twenty-three cases underwent an McKeown esophagectomy with cervical anastomosis, 8 cases underwent an Ivor-Lewis esophagectomy and 12 cases underwent a Sweet esophagectomy with intrathoracic anastomosis. Twenty-eight patients had esophageal cancer and 15 patients had esophagogastric junction cancer. Twenty-eight male and 15 female, age from 52 to 74, average age 64.5 ± 4.7. The stomach was mobilized and was used as a standard organ to reconstruct the continuity of the digestive tract in all patients. The anastomosis was performed via staplers in all 43 patients. The patients were divided into two groups according to the different treatments they received. 20 (47%) patients from January 2008 to December 2012 received conventional treatments (group 1), and 23 (53%) patients from January 2013 to December 2019 received new treatments (group 2), consisted of conventional therapies and the nasogastric placement of sump drainage tube through fistula into abscess cavity. Anastomotic leak was detected and confirmed by radiographic water-soluble iodine contrast esophagography when leak was under suspicion. The median time to confirmation of a significant leak was 6 days after surgery (range, 3–9 days). In all patients with intrathoracic anastomotic leaks, **conservative treatment strategies** were chosen, consisted of absence of oral intake, nasogastric suction drainage, enteral nutrition, antibiotic therapy, and drainage of the infected material through chest tube. This study was reviewed and approved by the institutional review board of the Hebei General Hospital.

The following patient characteristics were reviewed: age, gender, pathologic findings, chemoradiotherapy history, accompanying diseases, operation method, type of anastomosis location, timing of diagnosis and treatment of the leak, hospital mortality, and duration of hospitalization. The basic characteristics of the enrolled patients were shown in Table 1.

**Table 1:** The patients characteristics

Patients’ characteristics	Conventional treatments (group 1) (*n* = 20)	New treatments (group 2) (*n* = 23)	*P* value
Age	64.2 ± 4.8	65.1 ± 4.2	*P* = 0.259
Gender			
Male	12	16	*P* = 0.733
Female	8	7	
Cancer type			
Esophageal cancer	13	15	*P* = 0.780
Esophagogastric junction cancer	7	8	
Neoadjuvant chemotherapy	5	6	*P* = 0.936
Comorbidity			
Hypertension	6	7	*P* = 0.895
Diabetes	4	5	*P* = 0.936
Anastomosis location			
Cervical anastomosis	8	15	*P* = 0.818
Intrathoracic anastomosis	10	8	
Operation method			
McKeown esophagectomy	8	15	*P* = 0.611
Ivor-Lewis esophagectomy	4	4	
Sweet esophagectomy	6	4	
Timing of diagnosis	6.3	5.8	*P* = 0.09

### Operation and postoperative care

**Fig. 1:**
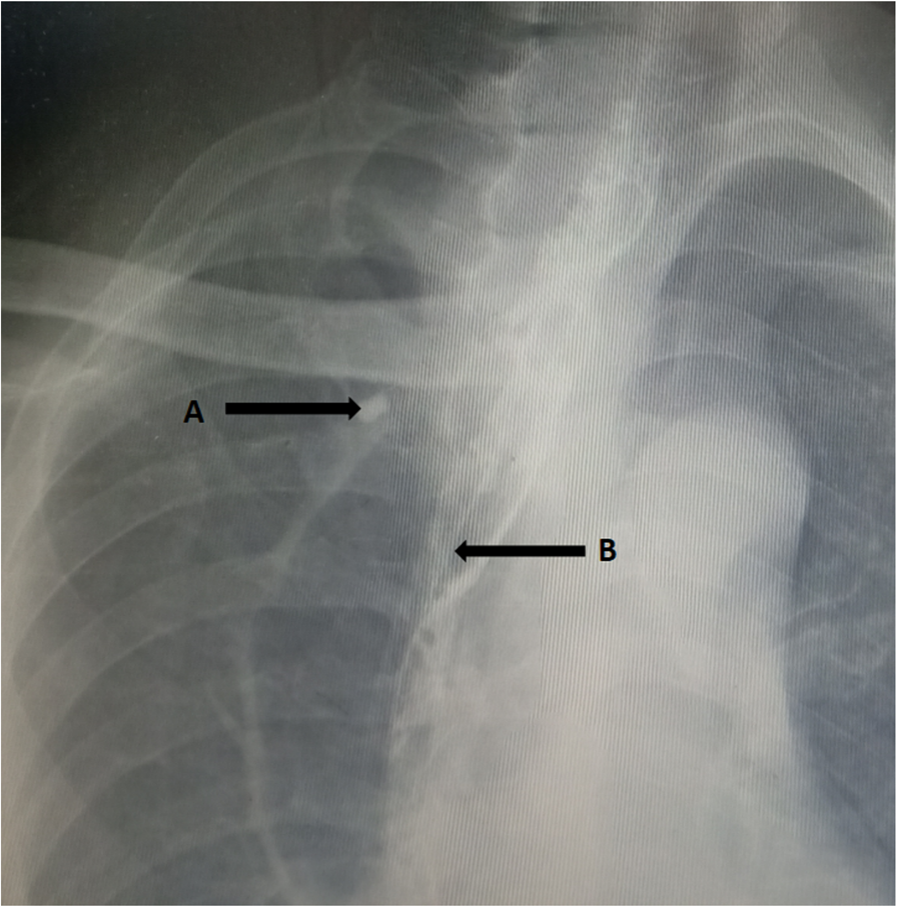
The image of contrast esophagography

**Fig. 2:**
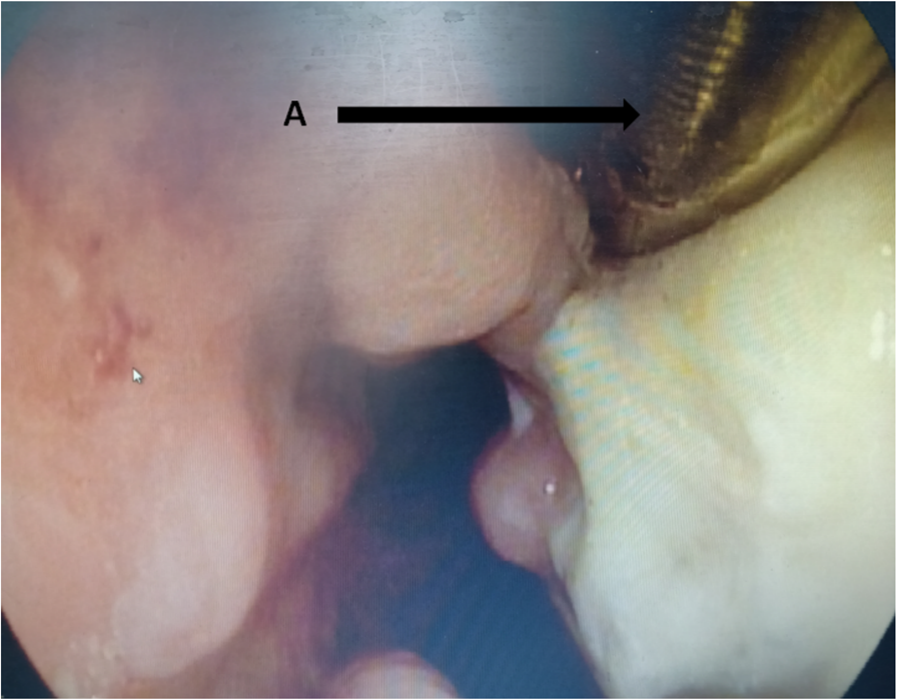
The gastroscopy after removal of sump drainage tube. **a** Nasogastric sump drainage tube (**a**) was inserted through the fistula with the guidance of radiography or gastroscopy and the distal tip of the tube was positioned at the bottom of the abscess cavity. **b** Naso-jejunum three lumen feeding tube (**b**) was placed under the radiographic or gastroscopic guidance. The functions of this tube include nasogastric decompression and nasojejunum enteral nutrition. **c** The closed fistula

For all patients, esophagectomy and esophagogastric anastomosis were performed. We used a circular mechanical stapler for anastomosis. A nasogastric decompression tube and thoracic and mediastinal drainage tubes were routinely placed during the procedure. The intubated patients were monitored in intensive care unit postoperatively. Following extubation, all patients stayed in intensive care unit for at least 24 h. The patients who were stable were transferred to our thoracic surgical ward. All patients were treated with fasting, gastrointestinal decompression, parenteral nutrition, antacids, and broad-spectrum antibiotics. If the clinical situation of the patient was stable, Water-soluble iodine contrast esophagography wloud be routinely performed on the 5th–7th postoperative day. If purulent drainage, fever, dyspnea, empyema, or wound infection possibly related to fistula were observed, Water-soluble iodine contrast esophagography was given immediately in order to confirm anastomosis leakage. When anastomotic leak was confirmed, all patients were placed with naso-jejunum three lumen feeding tube under the radiographic or gastroscopic guidance. This tube not only ensures enteral nutrition, but also provides gastrointestinal decompression (Figs. 1b; 2b). If there was no fever, leukocytosis, dyspnea, purulent drainage, the chest tube was removed. The mediastinal drainage tube was not removed until oral feeding. All patients were followed up for at least 6 months in the outpatient department.

### Conventional managements (group 1)

20 (47%) patients from January 2008 to December 2012 received conventional treatments (group 1). when intrathoracic anastomosis leak was confirmed, naso-jejunum three lumen feeding tube was placed under the radiographic or gastroscopic guidance. The functions of this tube include nasogastric decompression and nasojejunum enteral nutritional support. The chest drainage tubes should usually be placed under the guidance of ultrasonography. These patients were treated by the traditional “three-tube method”, **consisting of feeding tube, gastrointestinal decompression tube and chest drainage tube.** In addition, other treatments, such as fasting, broad spectrum antibiotics and antacids, were routinely performed. When the patients became stable, a contrast esophagography was performed every other week. Those patients whose leaks are confirmed to have been healed radiologically may start oral feeding.

### New managements (group 2)

**Fig. 3:**
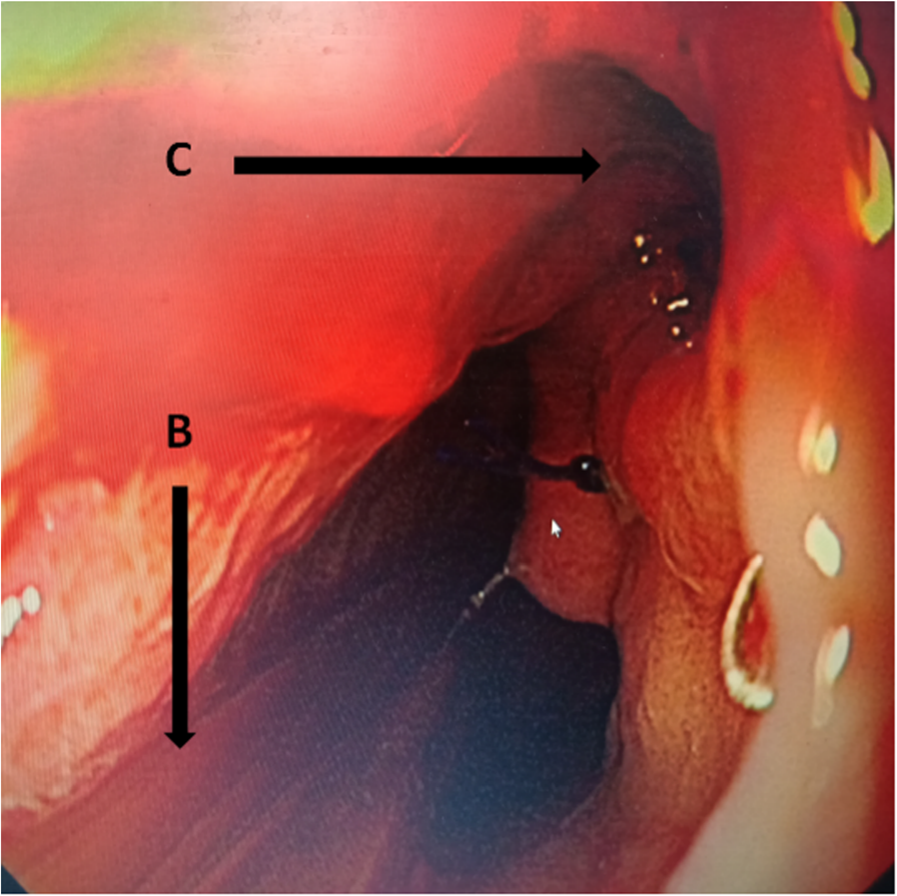
The gastroscopy during treatment

23 (53%) patients from January 2013 to December 2019 received new treatments (group 2). Intrathoracic anastomotic leak was identified by radiographic water-soluble iodine contrast esophagography when leak was under suspicion. In addition to the conventional treatments previously described, A sump drainage tube was introduced through the nose, remanet esophagus and inserted through the fistula into mediastinum with the guidance of radiography or gastroscopy. The distal tip of the sump drainage tube should be positioned at the bottom of the abscess cavity (Figs. 1a; 3a). Continuous suction with a negative pressure should be achieved for effective drainage. Contrast esophagography should be repeated once a week for treatment effect evaluation. The nasogastric sump drainage tube was used as the perianastomotic drain, which had multiple side holes. The drain position was checked under radiography by the injection of contrast medium through the tube. When the reduction of the cavity size was observed, the sump drainage tube was then retreated gradually. The sump drainage tube was pulled of 1–2 cm every 2–3 days until the tip of the tube was retreated to the esophageal cavity, the tube was removed. After termination of therapy, Contrast esophagography was performed once again to confirm the closure of the anastomotic leak. Thereafter, oral intake was gradually started.

### Statistical analysis

Statistical analysis was performed with SPSS version 21.0 software (IBM Corporation, Armonk, NY). Continuous variables were expressed as the means±SD, and categorical variables were expressed as percentages. A two independent samples t test was used for intergroup comparisons of the continuous variables, whereas X^2^ test or Fisher’s exact test was used to compare the categorical data. All tests were two-sided, *P* < 0.05 was considered statistically significant.

## Results

From January 2008 to December 2019, 875 patients received esophagectomy and esophagogastric anastomotic procedures in our department. The presence of intrathoracic anastomotic leak was confirmed by contrast esophagography in 43 patients (4.9%). Among them, 20 patients were treated by conventional treatments (group 1) known as the traditional “three-tube method”. Twenty-three patients were managed by new treatments (group 2), consisted of conventional therapies and the nasogastric placement of sump drainage tube through fistula into abscess cavity.

There were no significant differences in the mean age, gender, cancer type, neoadjuvant chemotherapy or comorbidities between the two groups (*P* > 0.05). The anastomosis location, operation method, and timing of diagnosis in the new treatments (group 2) were also similar compared with the conventional treatments (group 1) (*P* > 0.05). The details are summarized in Table 1. The postoperative data of the enrolled patients were shown in Table 2.

**Table 2:** Postoperative data collection

Postoperative observed indicators	Group 1 (*n* = 20)	Group 2 (*n* = 23)	*P* value
Duration of chest tube			
Average	47 days	28 days	*P* = 0.000
Range	36–54 days	23–42 days	
Length of leak treatment			
Average	52 days	35 days	*P* = 0.01
Range	40–61 days	28–52 days	
Length of postoperative hospital stay			
Average	56 days	39 days	*P* = 0.000
Range	45–70 days	32–54 days	
Hospital mortality	7 (35%)	3 (13%)	*P* = 0.148

### Duration of chest tube

The average duration of chest tube was 47 days in group 1 and 28 days in group 2, respectively. The minimum duration of chest tube was 36 days in group 1 and 23 days group 2, the maximum duration of chest tube was 54 days in group 1 and 42 days group 2, respectively.

### The length of leak treatment

The average length of leak treatment was 52 days (range, 40–61 days) in group 1 and 35 days (range, 28–52 days) in group 2, respectively.

### The length of postoperative hospital stay

The average length of postoperative hospital stay was 56 days, ranging from 45 to 70 days in group 1 and 39 days, ranging from 32 to 54 days in group 2, respectively.

### Hospital mortality

7 (35%) patients among 20 patients died from intrathoracic anastomotic leak in group 1; and 3 (13%) patients among 23 patients died from intrathoracic anastomotic leak in group 2.

Compared with the conventional treatments (group 1), The average duration of chest tube was significantly decreased in the new treatments (group 2) (*P* < 0.01), as well as the length of leak treatment (*P* < 0.05) and the length of postoperative hospital stay (*P* < 0.01). However, there was no significant difference in the mortality rate (*P* = 0.148 > 0.05; Table 2).

## Discussion

Intrathoracic esophagogastric anastomotic leak is one of the most serious complications after esophagectomy due to its associated high postoperative morbidity and mortality rates [15, 16]. In our study, the intrathoracic anastomotic leaks occurred in 4.9% of our patients, which was slightly lower than the reported incidence [13, 17]. This may be explained by our general treatment preference for staplers in the anastomosis, interrupted hand sutures for reinforcement.

Due to this wide clinical spectrum, the most effective treatment approach in introthoracic anastomotic leak is controversial. There is no standard treatment method especially for patients with symptomatic thoracic leaks. Gastric fluid has digestive properties that make leakage extremely noxious. Anaerobic bacteria from the patient’s oral cavity and swallowed saliva will cause a virulent tissue reaction and infection that can result in mediastinitis, empyema, or multiple organ failure. Generally speaking, fasting and decompressing the conduit with a nasogastric tube, parenteral or enteral nutrition and broad-spectrum antibiotics are basic steps once intrathoracic anastomotic leaks confirmed. Adequate drainage of infected fluid is much more necessary. This may include appropriately positioned chest drainage tubes to encourage full expansion of the lung. But it is often not enough, especially when pus is divided and mediastinitis is very serious. Leak from an anastomosis may lead to the formation of a perianastomotic abscess. So perianstomotic drain is very vital. The nasogastric sump drainage tube through fistula into abscess cavity was used as the perianastomotic drain under the guidance of radiography or gastroscopy, which could sufficiently drain purulent fluid nearby the anastomosis and prevent continuous contamination. Daily irrigation will be recommended when the abscess content is extremely viscid and thick. Maintenance of nutrition and complete drainage will allow granulation tissue to grow in this area and produce closure of the fistula. All patients were placed with naso-jejunum three lumen feeding tubes that not only ensure sufficient enteral nutrition, but also provide adequate gastrointestinal decompression in our study.

Conservative managements were usually performed in contained intrathoracic esophagogastric anastomotic leaks. The traditional “three-tube method” was the most widely applied method in Chinese thoracic clinics [18]. The principle of this method was effective chest tube drainage. Usually, a 24F chest tube was placed into the abscess under the guidance of CT scan or ultrasonography. When the abscess was limited in the pleural cavity, this method could achieve adequate drainage. But previous studies had reported that most of the contained leaks were limited to the mediastinum [13, 15]. It is difficult to deal with this type of leakage because abscess cavities close to the mediastinum are difficult to reach by the conventional chest tube. The nasogastric placement of sump drainage tube through the fistula in our study may be a helpful treatment for this type of leak. We successfully applied the nasogastric placement of sump drainage tube through the fistula for the treatment of intrathoracic anastomotic leaks in 23 patients. Compared with the conventional treatments of “three-tube method,” this management could achieve better drainage due to precise positioning and continuous negative pressure suction. Our study showed that the average length of leak treatment was 52 days in group 1 and 35 days in group 2. The average length of postoperative hospital stay was 56 days in group 1 and 39 days in group 2.Statistical analysis revealed that the treatment of nasogastric placement of sump drainage tube through the fistula may result in significantly shortened the length of leak treatment and hospitalization. In addition, these two treatment groups, mentioned above, had similar mortality rates according to our clinical results, ranging from 13 to 35%, which were considered acceptable according to previous studies [19, 20].

## Conclusion

In conclusion, Our results showed this method of the nasogastric placement of sump drainage tube through fistula appears to be an safe, effective, technically feasible treatment option for intrathoracic esophagogastric anastomotic leak. The efficacy and feasibility could be further investigated with a well-designed and large-scale RCT research.

## Data Availability

The datasets used and analysed during the current study are available from the corresponding author on reasonable request.

## Abbreviations

CT: Computed Tomography
SD: Standard Deviation
RCT: Randomized Controlled Trail
